# Supplementary material for: Oligoclonal CD4+CXCR5+ T cells with a cytotoxic phenotype appear in tonsils and blood
Source: Commun Biol. 2024 Jul 18;7:879. doi: 10.1038/s42003-024-06563-1 (PMC11258247; doi:10.1038/s42003-024-06563-1)
Supplement: Supplementary file 2 — Supplementary information [file 42003_2024_6563_MOESM2_ESM.pdf]

Supplementary Information

Oligoclonal $CD4^+CXCR5^+$ T cells with a cytotoxic phenotype appear in tonsils and blood

Running title: Transcriptional landscape of T_{FH} subtypes

Chunguang Liang[1,10], Silvia Spoerl[2], Yin Xiao[3], Katharina M. Habenicht[4], Sigrun S. Haeusl[3], Isabel Sandner[2], Julia Winkler[2], Nicholas Strieder[5], Rüdiger Eder[6], Hanna Stanewsky[5], Christoph Alexiou[7], Diana Dudziak[8,10], Andreas Rosenwald[3,9], Matthias Edinger[5,6], Michael Rehli[5,6], Petra Hoffmann[5,6,11], Thomas H. Winkler[4,11], Friederike Berberich-Siebelt[3,11,*]

[1]Functional Genomics and Systems Biology Group, Department of Bioinformatics, Biocenter, Julius-Maximilians-University Würzburg; Würzburg, Germany.

[2]Department of Internal Medicine 5, Hematology/Oncology, University Hospital Erlangen, Friedrich-Alexander-University Erlangen-Nürnberg (FAU), Comprehensive Cancer Center Erlangen-EMN; Erlangen, Germany.

[3]Institute of Pathology, Julius-Maximilians-University Würzburg; Würzburg, Germany.

[4]Division of Genetics, Department Biology, Nikolaus-Fiebiger-Center of Molecular Medicine, Friedrich-Alexander University Erlangen-Nürnberg; Erlangen, Germany.

[5]Leibniz Institute for Immunotherapy; Regensburg, Germany.

[6]Department of Internal Medicine III, University Hospital Regensburg; Regensburg, Germany.

[7]Department of Otorhinolaryngology, Head & Neck Surgery, Else Kröner-Fresenius-Foundation-Professorship, Section of Experimental Oncology & Nanomedicine (SEON), University Hospital Erlangen, Friedrich-Alexander University Erlangen-Nürnberg; Erlangen, Germany.

[8]Laboratory of Dendritic Cell Biology, Department of Dermatology, University Hospital Erlangen, Friedrich-Alexander University of Erlangen-Nürnberg; Erlangen, Germany.

[9]Comprehensive Cancer Centre Mainfranken, Julius-Maximilians-University of Würzburg; Würzburg, Germany.

[10]Present address: Institute of Immunology, Jena University Hospital, Friedrich-Schiller-University, Jena, Germany.

[11] These authors jointly supervised this work: Petra Hoffmann, Thomas H. Winkler, Friederike Berberich-Siebelt.

* Corresponding author: Prof. Dr. Friederike Berberich-Siebelt

Institute of Pathology, University of Würzburg, Josef-Schneider-Str. 2, 97080 Würzburg, Germany

Phone: +49 931 31 81208; fax: +49 931 81224; e-mail: path230@mail.uni-wuerzburg.de

Supplementary Table 1: Healthy and patient donors

Patients under cyclosporine A after allogeneic-HSCT

Code		age recipient	sex recipient	conditioning regimen prior to allo-HSCT	Age donor	sex donor	Time after CsA tapering	GvHD
**CsA 1a/b**	**CsA1a:** before CsA tapering<br>**CsA1b:** after CsA tapering	39	female	busulfane, cyclophosphamide	35	male	5 weeks	no
**CsA 2a/b**	**CsA2a**: before CsA tapering<br>**CsA2b:** after CsA tapering	67	male	fludarabine, busulfane, melphalane	33	male	3 weeks	no

Vaccinated healthy donors

Code		age	sex	type of vaccination		Time from last vaccine to booster	Analysis after vaccination
**Vac 1a/b**	**Vac1a:** before vaccination<br>**Vac1b**: after vaccination	40	female	active (tetanol)		10 years	7 days
**Vac 2a/b**	**Vac2a:** before vaccination<br>**Vac2b**: after vaccination	33	male	active (tetanol)		13 years	7 days

Patients treated with tonsillectomy

Code		age	sex	Present tonsillitis	% T_{FK} of T_{FH}	Analysis
**Ton1**		4	female	no	n.d.	scRNAseq
**Ton2**		30	female	yes, moderate	n.d.	scRNAseq
**Ton3**		5	male	yes, mild	n.d.	scRNAseq
**Ton4**		25	female	yes, mild	>3	Flow cytometry / IF histology
**Ton5**		5	female	yes, mild	<1	Flow cytometry / IF histology
**Ton6**		5	female	yes, mild	<1	Flow cytometry / IF histology
**Ton7**		4	male	yes, mild	>3	Flow cytometry / IF histology
**Ton8**		16	male	yes, mild	<1	Flow cytometry / IF histology
**Ton9**		35	male	yes, mild	>3	Flow cytometry / IF histology
**Ton10**		3	male	no	<1	Flow cytometry / IF histology
**Ton11**		3	female	yes, mild	<1	Flow cytometry / IF histology

13 anonymous healthy adult volunteers - blood donors for flow cytometry

Supplementary Data 1: see excel file

Supplementary Table 2: Antibodies used for flow cytometric sorting

Antigen	Clone	Dilution	Conjugate	Vendor + Cat #
**CD3**	SK7	1:20	BV510	BD Biosciences 569250
**CD4**	SK3	1:10	PE	BD Biosciences 565999
**CD45RA**	L48	1:10	FITC	BD Biosciences 335039
**CXCR5**	RF8B2	1:20	AF647	BD Biosciences 558113
**CD25**	BC96	1:50	TotalSeq™-C0085	BioLegend 302649
**CD279**	EH122H7	1:50	TotalSeq™- C0088	BioLegend 329963
**CD183**	G025H7	1:50	TotalSeq™-C00140	BioLegend 353747
**CD196**	G034E3	1:50	TotalSeq™-C00143	BioLegend 353445

Supplementary Table 3: Antibodies used for flow cytometric analysis

Antigen	Clone	Dilution	Fluorochrome	Vendor + Cat #
**CD3**	SK7	1:50	BUV395	BD Biosciences 564001
**CD3**	OKT3	1:50	AF700	BioLegend 317340
**CD4**	SK3	1:20	FITC	BioLegend 344604
**CD4**	SK3	1:50	Spark NIR 685	BioLegend 344658
**CD8a**	SK1	1:40	BUV805	BD Biosciences 612889
**CD8b**	REA715	1:100	VioGreen	Miltenyi Biotec 130-110-516
**CD19**	SJ25C1	1:40	BUV737	BD Biosciences 612756
**CD45RA**	HI100	1:100	BV711	BD Biosciences 563733
**CD45RA**	HI100	1:20	PerCP	BioLegend 304156
**CD56**	NCAM16.2	1:100	BUV615	BD Biosciences 613001
**CD107a**	H4A3	1:20	BV786	BD Biosciences 563869
**CXCR5**	RF8B2	1:20	AF647	BD Biosciences 558113
**CXCR5**	J252D4	1:20	APC	BioLegend 356908
**EOMES**	WD1928	1:50	PE-Cy7	Invitrogen, Thermo Fisher 25-4877-42
**Granzyme B**	GB11	1:20	BV421	BD Biosciences 563389
**TIA-1**	2G9	1:100 /150	PE	Beckman Coulter IM3293
**PD-1**	EH12.2H7	1:20	Dazzle 594	BioLegend 329940
**CXCR3**	G025H7	1:50	BV510	BioLegend 353726
**CCR6**	G034E3	1:20	BV650	BioLegend 353426
**CD57**	QA17A04	1:80	BV711	BioLegend 393328

Supplementary Table 4: Antibodies used for IF histology staining

Antigen	Clone	Dilution	Fluorochrome	Vendor + Cat #
**CD4**	Polyclonal Goat IgG	1:150	-	R&D AF-379-NA
**TIA-1**	2G9A10F5	1:200	-	Beckman Coulter IM2550
**BCL-6**	D4I2V	1:50	-	Cell signaling 14895
**CD19**	6OMP31	1:500	-	Invitrogen 14-0194-82
**IgD**	Polyclonal Goat IgG	1:100	-	Southern Biotech 2032-01
**CD23**	1B12	1:250	-	Leica NCL-L-CD23-1B12
**Ki67**	SolA15	1:200	-	Invitrogen 14-5698-82
**CXCR5**	EPR23463-30	1:300	-	Abcam ab254415
**Hoechst**	-	1:50000	-	Sigma B2261
donkey anti-goat	-	1:1000	Alexa Fluor 594	Abcam ab150132
donkey anti-rabbit	-	1:1000	Alexa Fluor 555	ThermoFisher A31572
donkey anti-rat	-	1:1000	Alexa Fluor 488	ThermoFisher A21208
donkey anti-mouse	-	1:1000	Alexa Fluor 647	ThermoFisher A31573
donkey anti-goat	-	1:1000	Alexa Fluor 546	ThermoFisher A11056

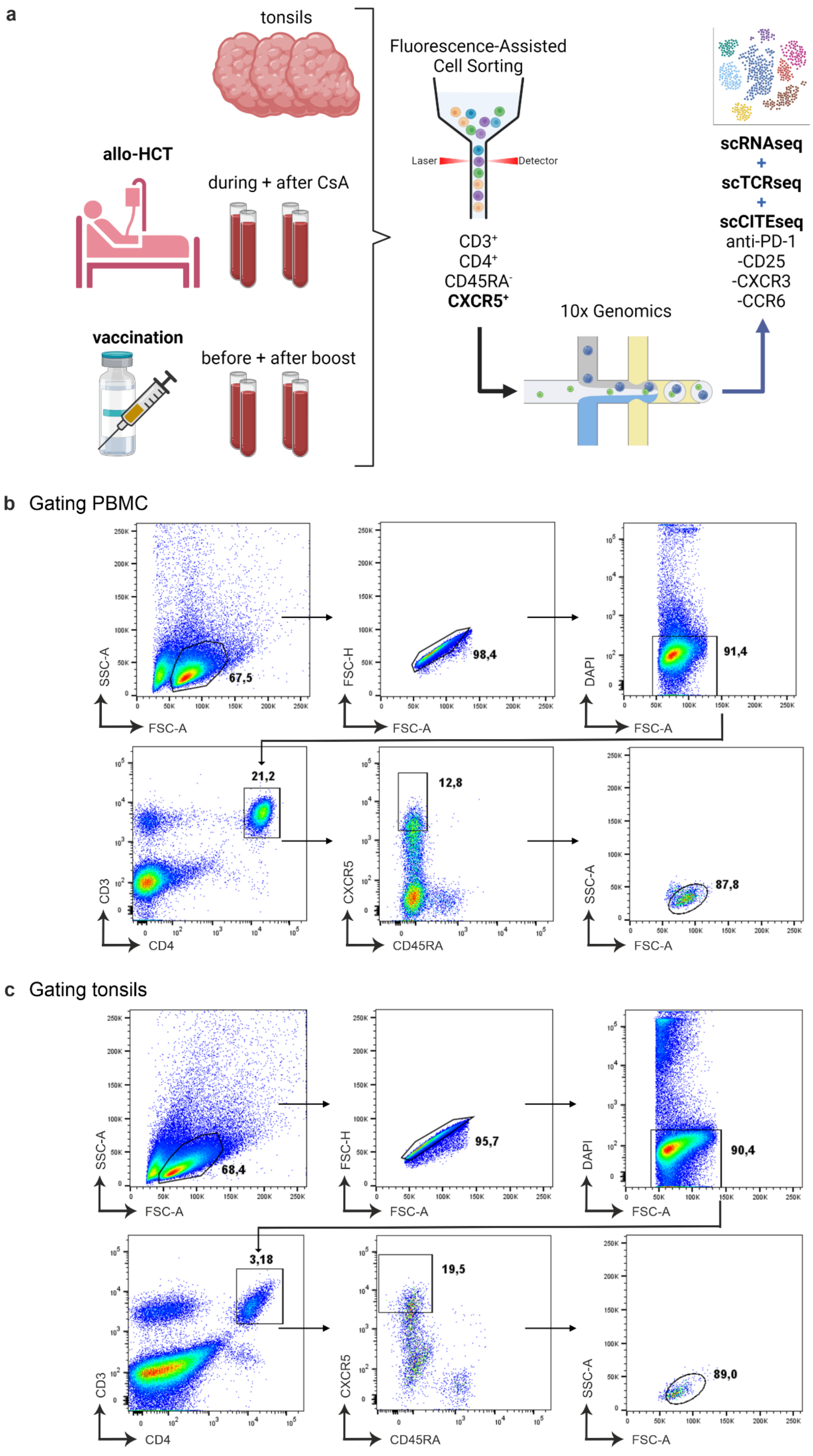
a
tonsils
allo-HCT
during + after CsA
vaccination
before + after boost
Fluorescence-Assisted
Cell Sorting
Laser
Detector
CD3+
CD4+
CD45RA-
CXCR5+
10x Genomics
scRNAseq
+
scTCRseq
+
scCITEseq
anti-PD-1
-CD25
-CXCR3
-CCR6
b Gating PBMC
SSC-A
FSC-A
67,5
FSC-H
98,4
DAPI
91,4
CD3
CD4
21,2
CXCR5
CD45RA
12,8
87,8
c Gating tonsils
68,4
95,7
90,4
3,18
19,5
89,0

Supplementary Fig. 1: Experimental design and gating strategy for T_{FH} cell sorting. a Lymphocytes from three tonsils (2x rhonchopathies with no or limited signs of inflammation and 1x recurrent cryptic tonsillitis), from PB of two allo-HSCT patients without signs of graft-versus-host disease (GvHD), during CsA treatment and two or three weeks after CsA tapering as well as from PBMCs of two healthy donors before and one week after booster vaccination were flow cytometry-sorted. All live (DAPI^{-}) CD3^{+}CD4^{+}CD45RA^{-}CXCR5^{+} T cells, additionally labeled with oligo-coupled anti-PD-1, -CD25, -CXCR3 and -CCR6, were subjected to single-cell RNA sequencing (scRNAseq). scRNAseq, scTCRseq and scCITEseq libraries were sequenced by 10x Genomics. **Created with** BioRender.com **b-c** Gating strategy for the sorting of T_{FH} cells identified as single living cells with the phenotype CD3^{+}CD4^{+}CXCR5^{+}CD45RA^{-} from PB **b** and tonsil samples **c**.

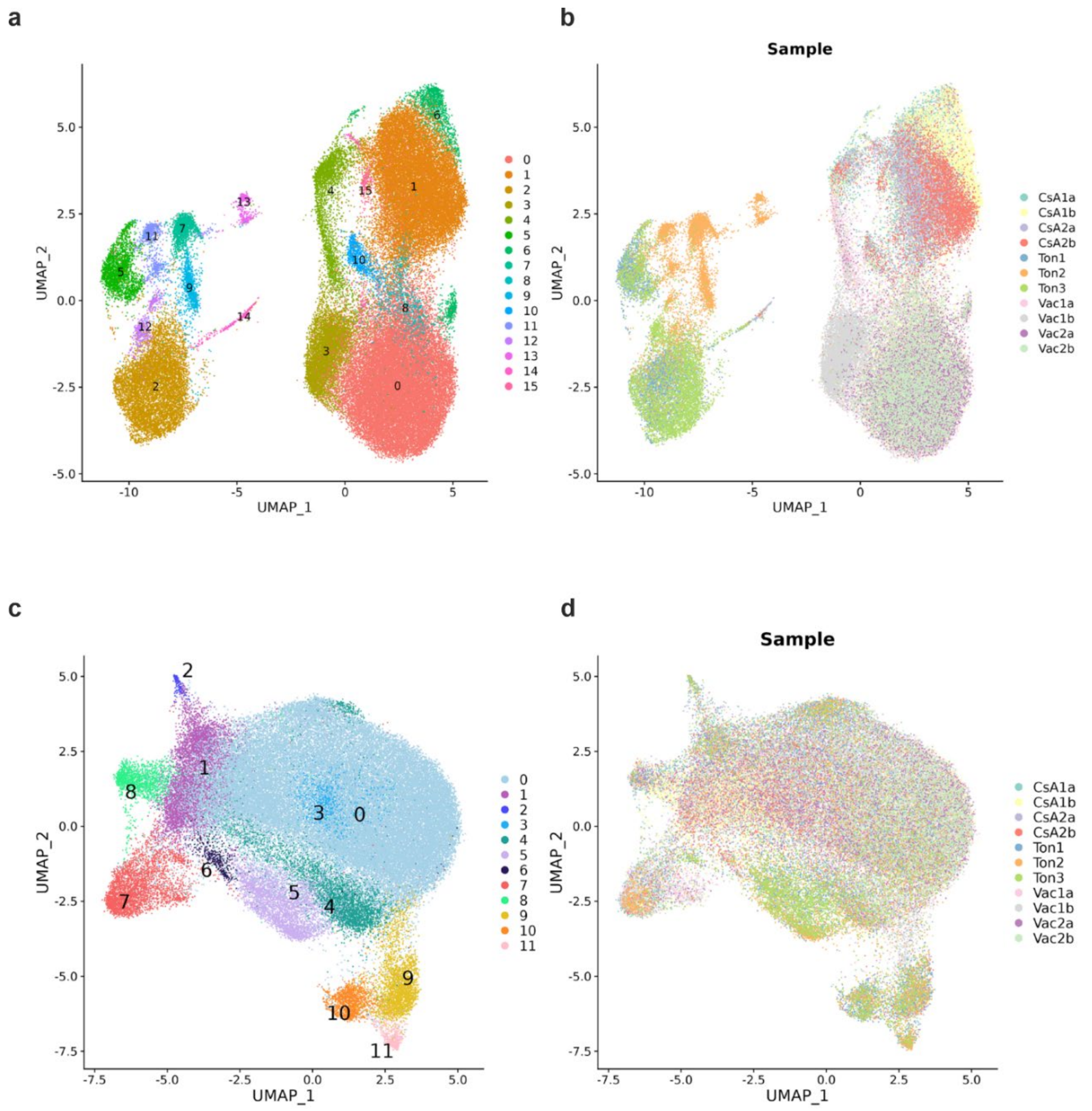

Supplementary Fig. 2: Seurat-CCA diminishes the batch-effect. a 16 cell clusters before minimizing the batch-effect, **b** originating from different libraries. All tonsillar cells are located in the left island (orange, light green and blue dots) and most of CsA cells and Vac cells are also separated from each other in the right island. **c** 12 cell clusters after minimizing the batch-effect by Seurat-CCA (dual PCA). Anchor genes were determined for data integration, followed by FindNeighbors, RunPCA and FindClusters. **d** The remaining smaller batch effect stems from those cells that are extremely enriched in either tonsil or blood samples.

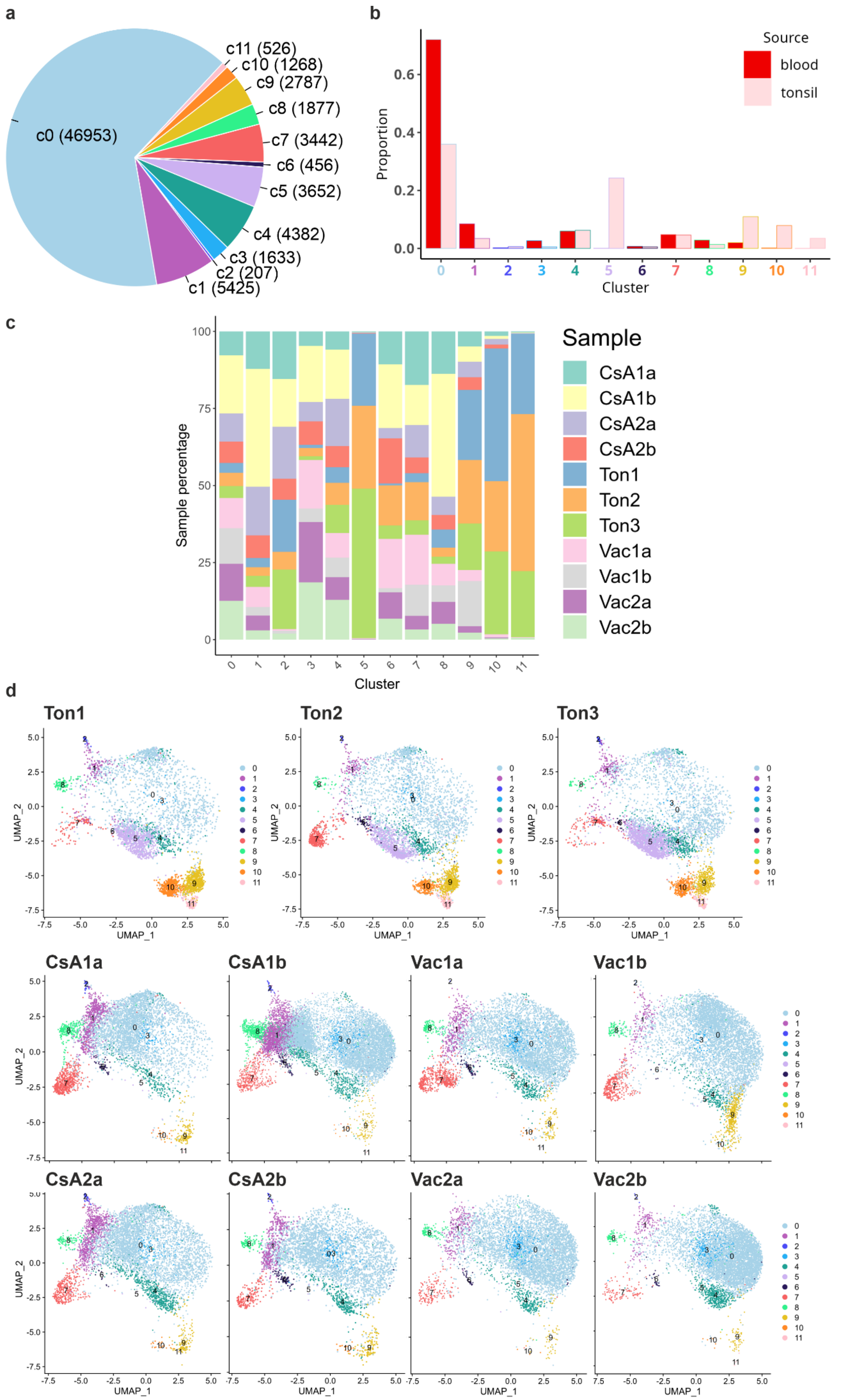
a
c0 (46953)
c11 (526)
c10 (1268)
c9 (2787)
c8 (1877)
c7 (3442)
c6 (456)
c5 (3652)
c4 (4382)
c3 (1633)
c2 (207)
c1 (5425)
b
Source
blood
tonsil
Proportion
Cluster
c
Sample
CsA1a
CsA1b
CsA2a
CsA2b
Ton1
Ton2
Ton3
Vac1a
Vac1b
Vac2a
Vac2b
Sample percentage
Cluster
d
Ton1
Ton2
Ton3
CsA1a
CsA1b
Vac1a
Vac1b
CsA2a
CsA2b
Vac2a
Vac2b
UMAP_1
UMAP_2

Supplementary Fig. 3: Relative distribution of $CD3^+CD4^+CXCR5^+$ clusters is different in PB and tonsils. **a** Pie chart for the number of cells and the relative contribution of each cluster in all sequenced cells. **b** Bar graph for the relative contribution of each cluster within PBMCs *vs* tonsils. **c** Bar graph to depict the relative sample abundance per cluster; colors represent the respective origin of the sorted cells. **d** Individual UMAP for cells of all different origins.

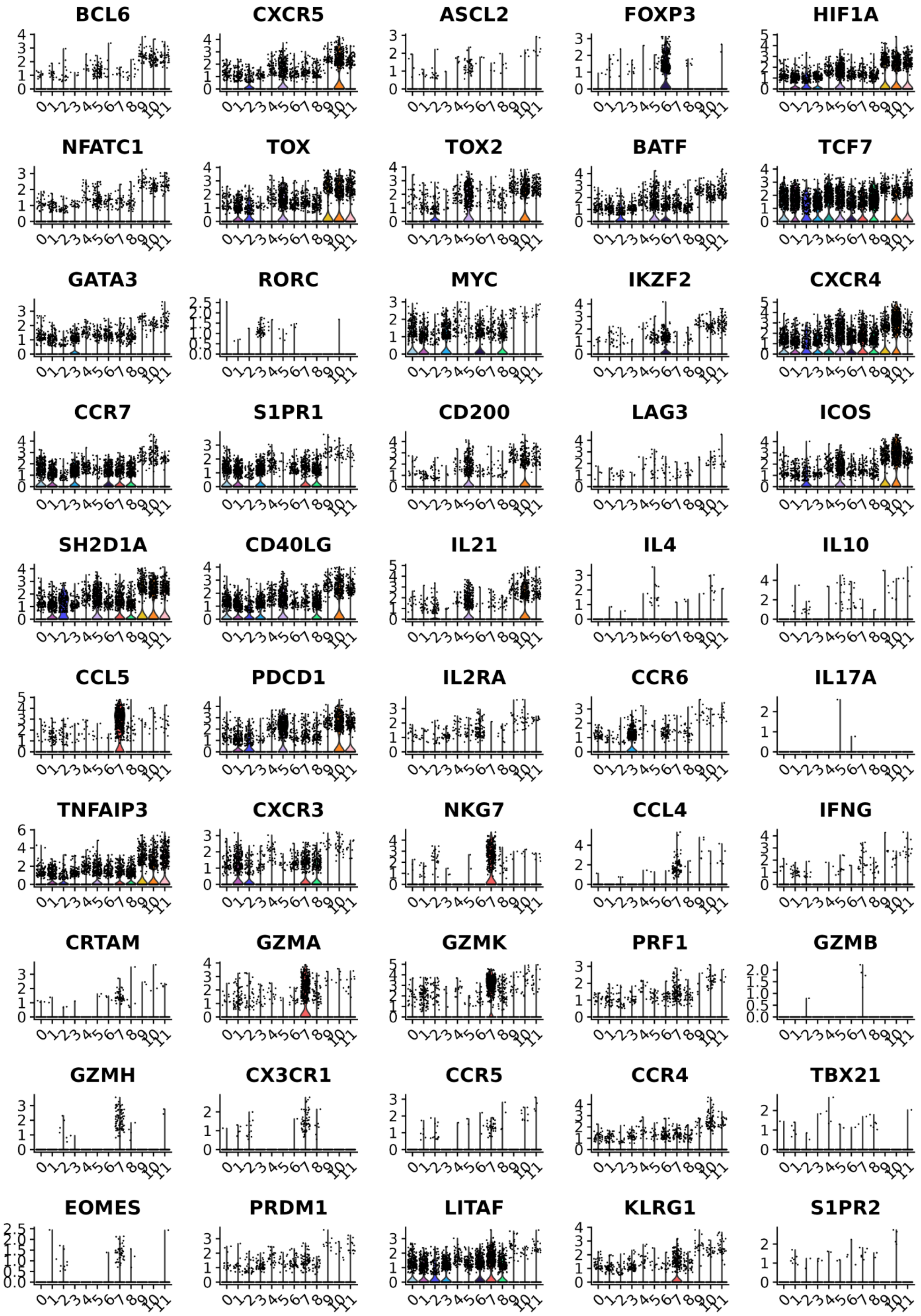

Supplementary Fig. 4: Violin plots for chosen transcripts illustrating the quantitative gene expression difference in 12 cell clusters. Color code corresponds to the previous UMAP.

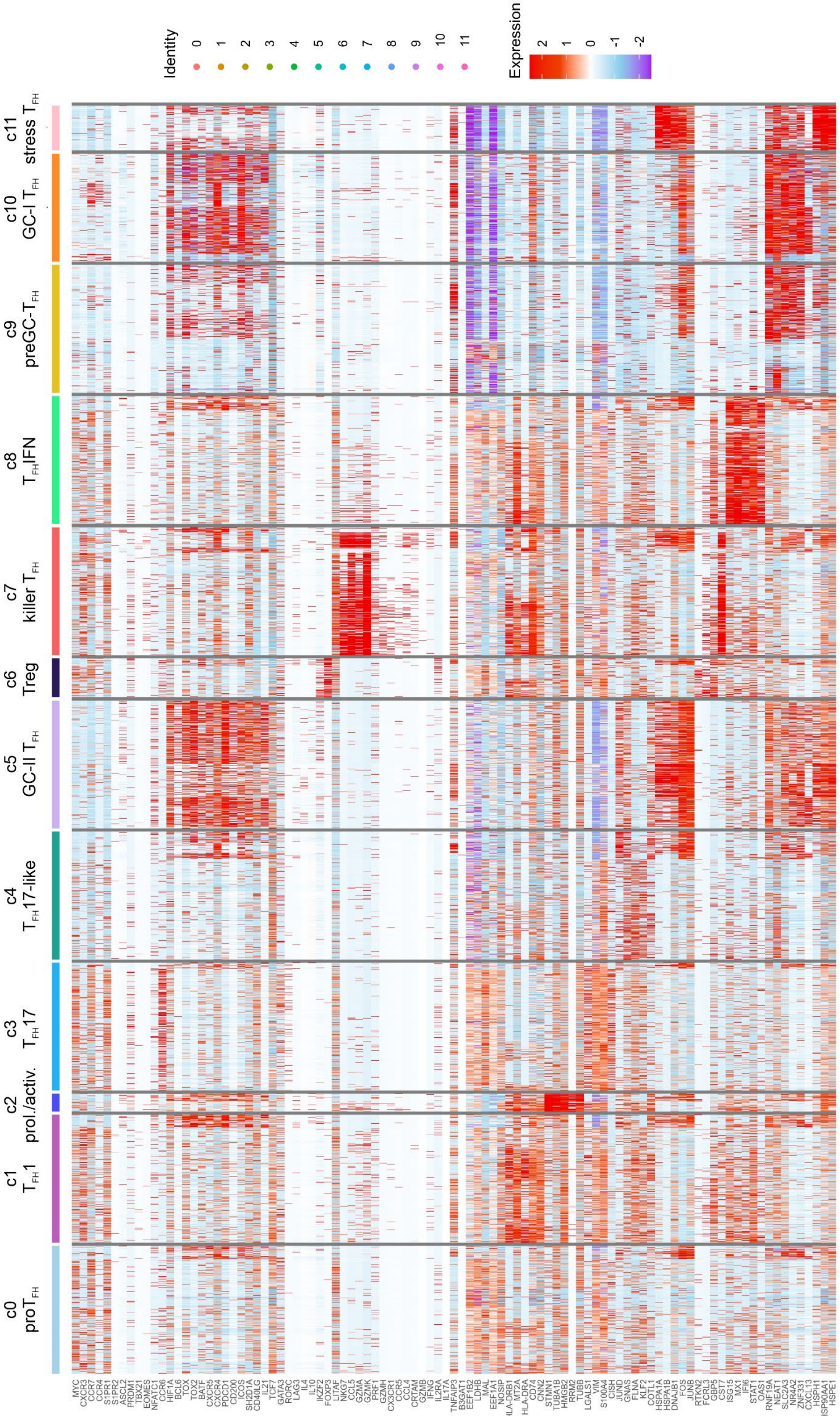
Identity
0
1
2
3
4
5
6
7
8
9
10
11
Expression
2
1
0
-1
-2
c11 stress T_{FH}
c10 GC-I T_{FH}
c9 preGC-T_{FH}
c8 T_{FH}IFN
c7 killer T_{FH}
c6 Treg
c5 GC-II T_{FH}
c4 T_{FH}17-like
c3 T_{FH}17
c2 prol./activ.
c1 T_{FH}1
c0 proT_{FH}

Supplementary Fig. 5: Heatmap depicting gene markers for each cell cluster. The upper part are selected markers which are known to be able to distinguish cell types such as Tregs, activated T cells, and T_{FH}, including cytokines, cytokine receptors and transcription factors. The lower part depicts the top 5 gene markers identified by Seurat differential gene expression analysis using a Wilcox test method. The scaled data are used in the illustration, where red indicates that the gene is strongly expressed, while blue represents low expression in the cell.

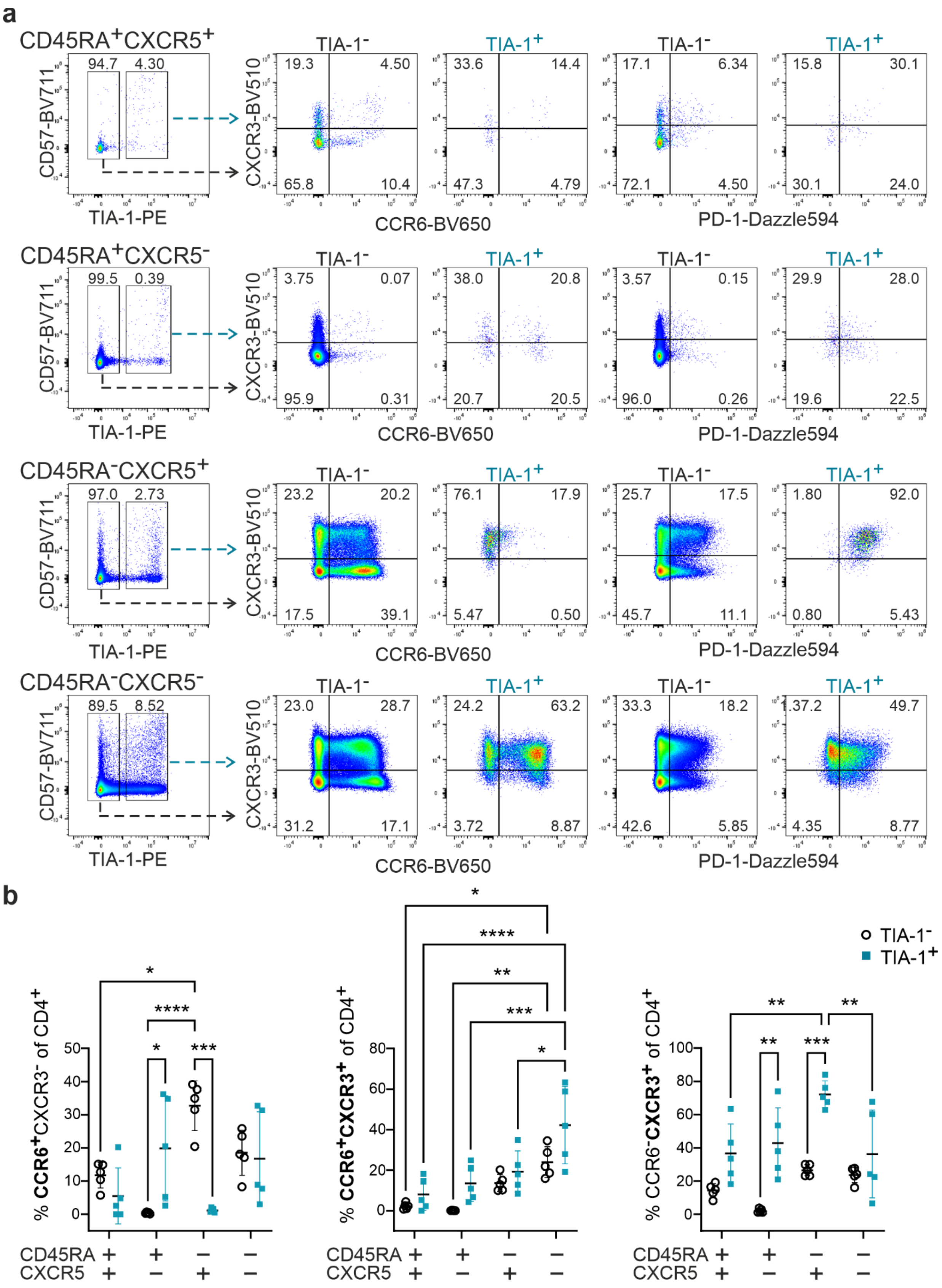
a
CD45RA+CXCR5+
CD45RA+CXCR5-
CD45RA-CXCR5+
CD45RA-CXCR5-
CD57-BV711
TIA-1-PE
CXCR3-BV510
CCR6-BV650
PD-1-Dazzle594
TIA-1-
TIA-1+
b
% CCR6+CXCR3- of CD4+
% CCR6+CXCR3+ of CD4+
% CCR6-CXCR3+ of CD4+
CD45RA
CXCR5
TIA-1-
TIA-1+

Supplementary Fig. 6: CXCR3, but not CCR6 correlates with TIA-1 expression, corresponding to Fig. 3 c-d**.** Leukocytes isolated from PB by Ficoll, stained immediately and gated for $CD3^+CD4^+CD45RA^-$ or $CD45RA^+$ and $CXCR5^-$ or $CXCR5^+$. **a** Frequencies of CXCR3 *vs* CCR6 and CXCR3 *vs* PD-1 of $TIA\text{-}1^-$ and $TIA\text{-}1^+$ cells are depicted in representative dot plots for $CD45RA^+CXCR5^+$, $CD45RA^+CXCR5^-$, $CD45RA^-CXCR5^+$ T_{FH} (as in Fig. 3c), and $CD45RA^-CXCR5^-$ cells. **b** $CCR6^+CXCR3^-$, $CCR6^+CXCR3^+$, and $CCR6^-CXCR3^+$ (≙ Fig. 3d) expression are demonstrated for $TIA\text{-}1^-$ and $TIA\text{-}1^+$ $CD4^+$ $CD45RA^{+/-}$ $CXCR5^{+/-}$ cells shown as cumulative graphs (two-way ANOVA with Šídák post-hoc analysis, n=5, *: $p \leq 0.05$, **: $p \leq 0.01$, ***: $p \leq 0.001$, ****: $p \leq 0.0001$).

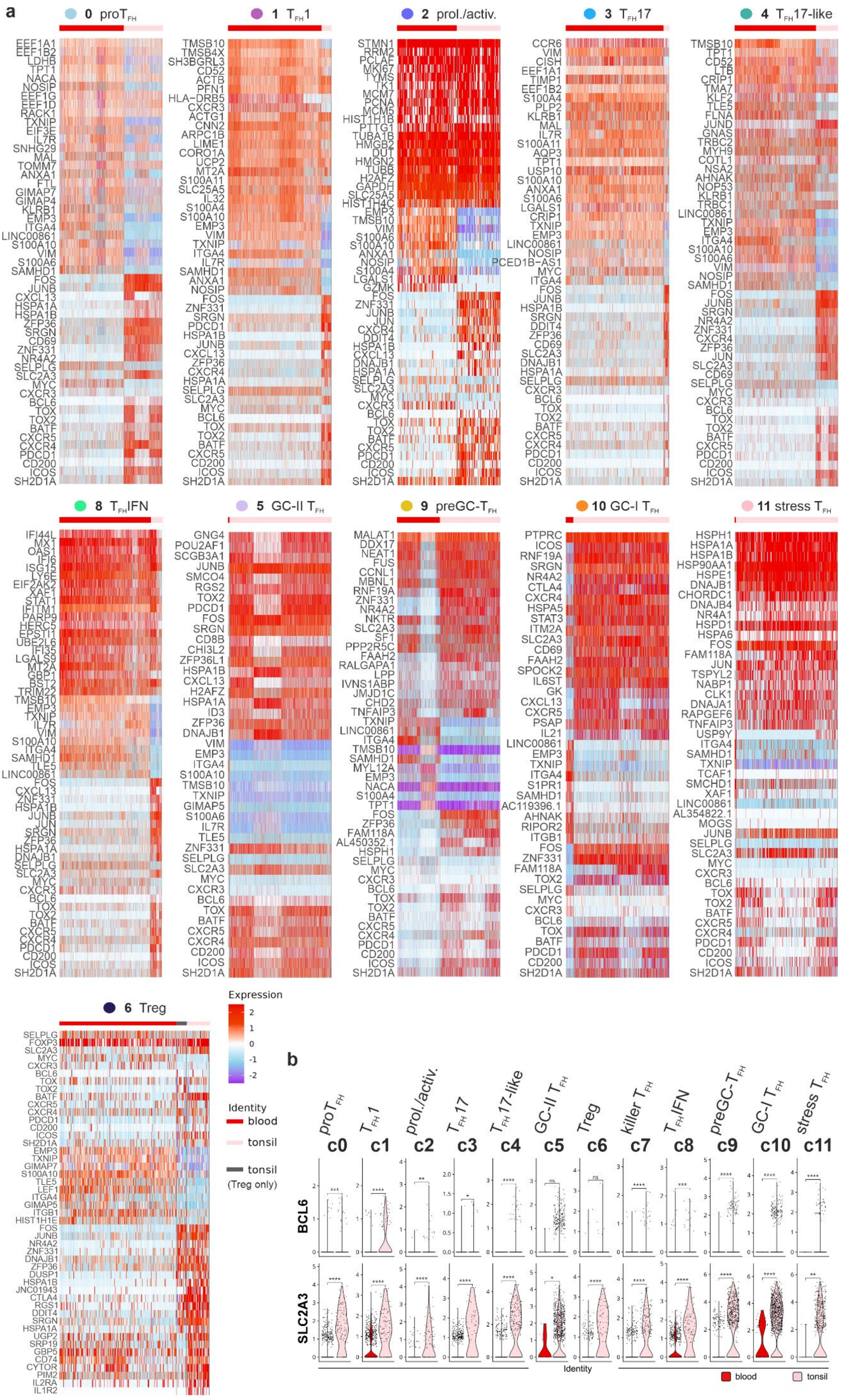
a
0 proT FH
1 T FH 1
2 prol./activ.
3 T FH 17
4 T FH 17-like
8 T FH IFN
5 GC-II T FH
9 preGC-T FH
10 GC-I T FH
11 stress T FH
6 Treg
Expression
2
1
0
-1
-2
Identity
blood
tonsil
tonsil
(Treg only)
b
proT FH
T FH 1
prol./activ.
T FH 17
T FH 17-like
GC-II T FH
Treg
killer T FH
T FH IFN
preGC-T FH
GC-I T FH
stress T FH
c0 c1 c2 c3 c4 c5 c6 c7 c8 c9 c10 c11
BCL6
SLC2A3
Identity
blood
tonsil

Supplementary Fig. 7: All $CD4^+CXCR5^+$ clusters show origin-specific gene expression. a Heatmaps of individual clusters, defined by the transcripts given in the upper parts, illustrate the differentially expressed genes, which can distinguish the 'blood' from the 'tonsil' cell subset. The top gene markers for both cell types are determined by a Wilcox test. **b** Violin plots showing *BCL6* and *SLC2A3* in each cluster. Dark red indicates the cell clusters enriched in blood samples, while dusky pink indicates those enriched in tonsil. A t-test was used to compare the gene expression levels between the two groups. ns: $p > 0.05$, *: $p \leq 0.05$, **: $p \leq 0.01$, ***: $p \leq 0.001$, ****: $p \leq 0.0001$.

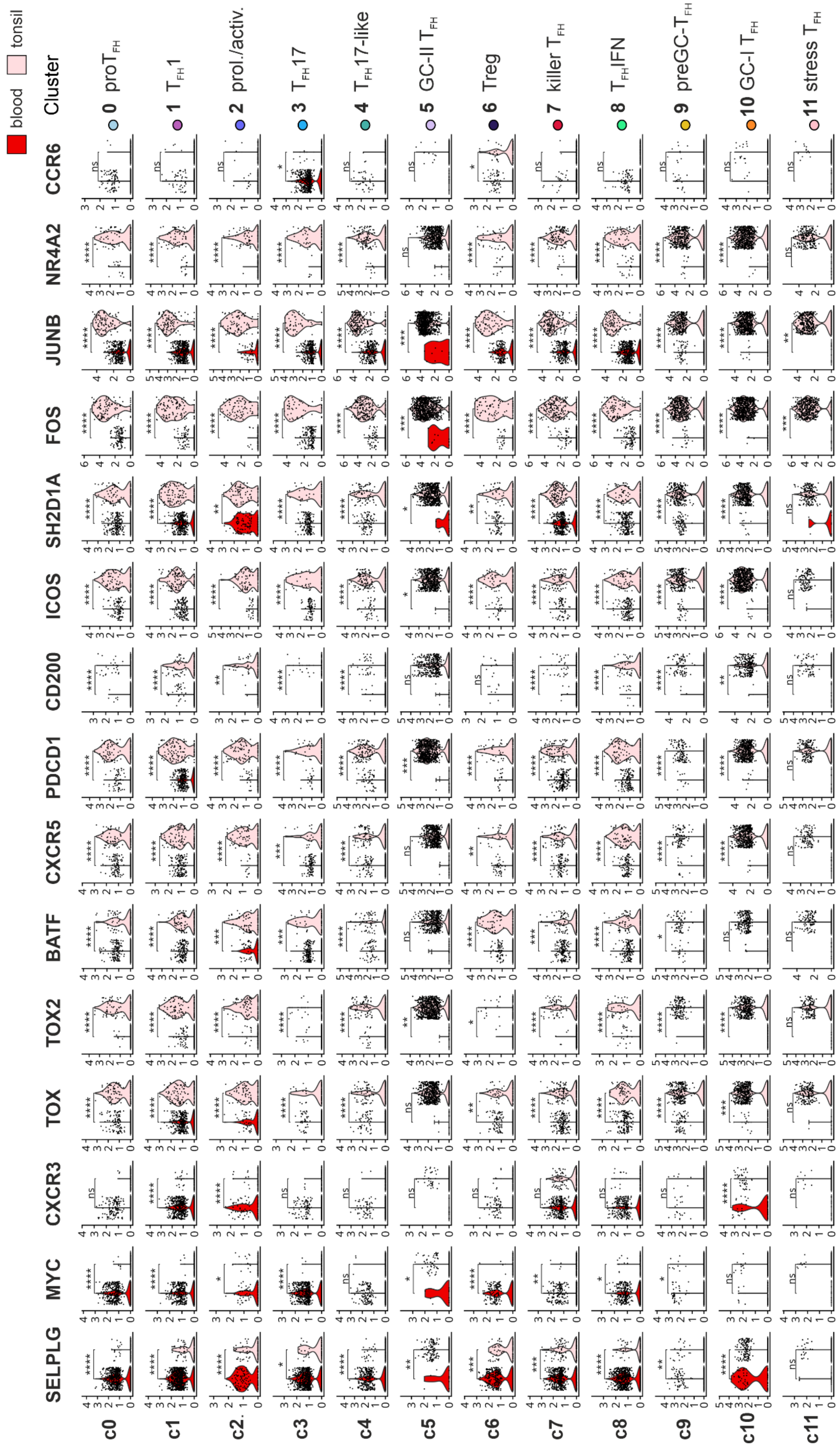
blood
tonsil
Cluster
0 proT_FH
1 T_FH1
2 prol./activ.
3 T_FH17
4 T_FH17-like
5 GC-II T_FH
6 Treg
7 killer T_FH
8 T_FHIFN
9 preGC-T_FH
10 GC-I T_FH
11 stress T_FH
CCR6
NR4A2
JUNB
FOS
SH2D1A
ICOS
CD200
PDCD1
CXCR5
BATF
TOX2
TOX
CXCR3
MYC
SELPLG
c0
c1
c2
c3
c4
c5
c6
c7
c8
c9
c10
c11

Supplementary Fig. 8: Within each cluster, classical T_{FH} genes are significantly more highly expressed in tonsillar cells. RNA expression of chosen genes is illustrated in violin plots and compared between tonsillar cells (dusty pink) and blood cells (red) for each cell cluster individually. Panels are aligned by gene names on the x-axis, and cell clusters/types on the y-axis. Symbols indicate the statistical significance: ns: $p > 0.05$, *: $p \leq 0.05$, **: $p \leq 0.01$, ***: $p \leq 0.001$, ****: $p \leq 0.0001$. A t-test is used to compare gene expression levels between two groups.

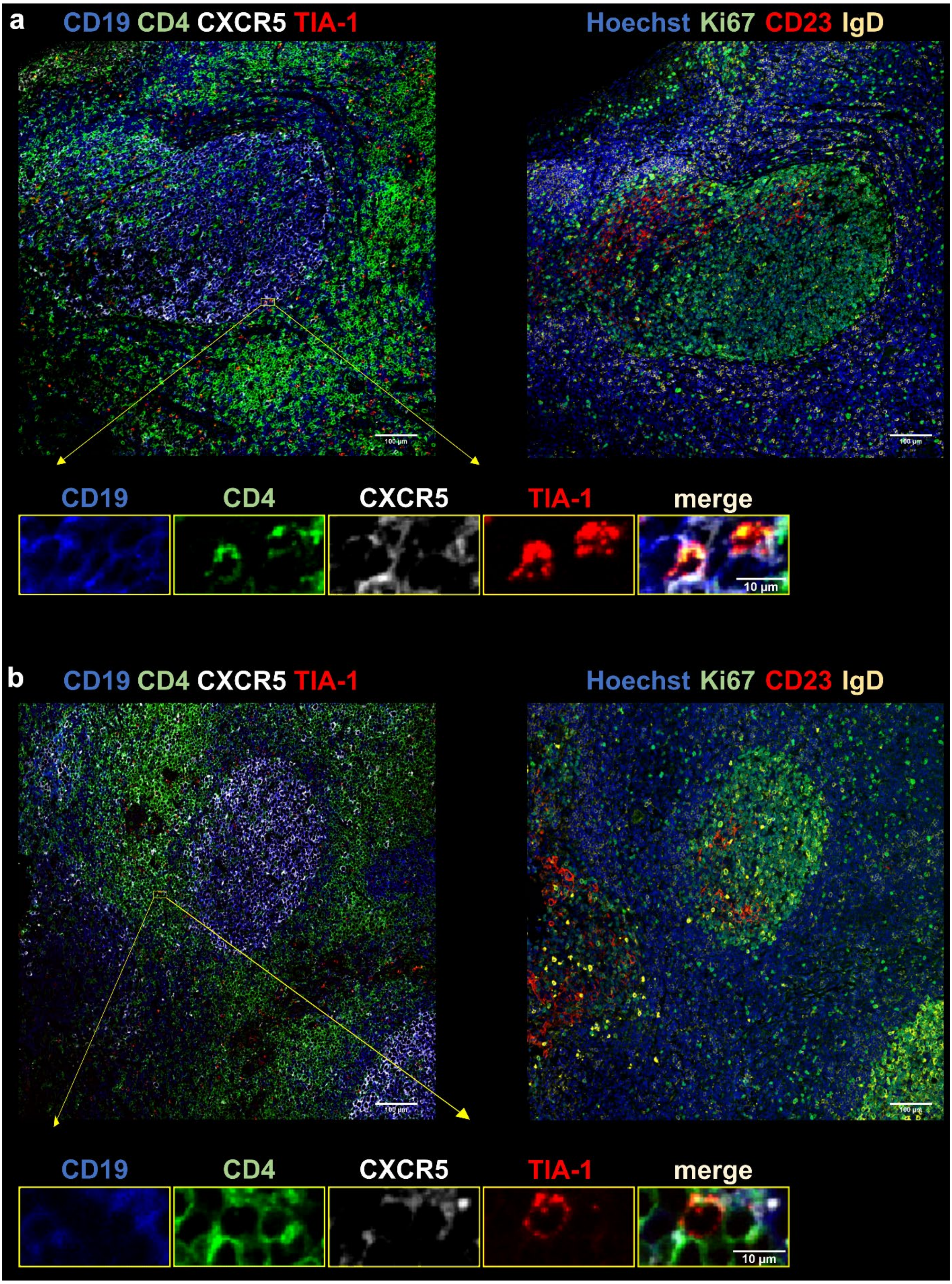

Supplementary Fig. 9: Only some CD4⁺CXCR5⁺TIA-1⁺ T cells localize at / within GCs. Two different tonsils with ≤ 1 % T_{FK} cells (Ton11; **a**) or ≥ 3 % (Ton7; **b**) as measured by flow cytometry.

Representative images of CD4 (green), CD19 (blue), CXCR5 (white), and TIA-1 (red; antibody for NKG7) staining patterns in a human tonsillar FFPE tissue section (left panel), and GC-B cells (Hoechst/blue, Ki67/green, CD23/red and IgD/yellow; scale bars = 100 µm). Enlarged images of one T_{FK} cell each with anti-CD19, -CD4, -CXCR5, -TIA-1 (NKG7) are depicted below, scale bars = 10 µm.

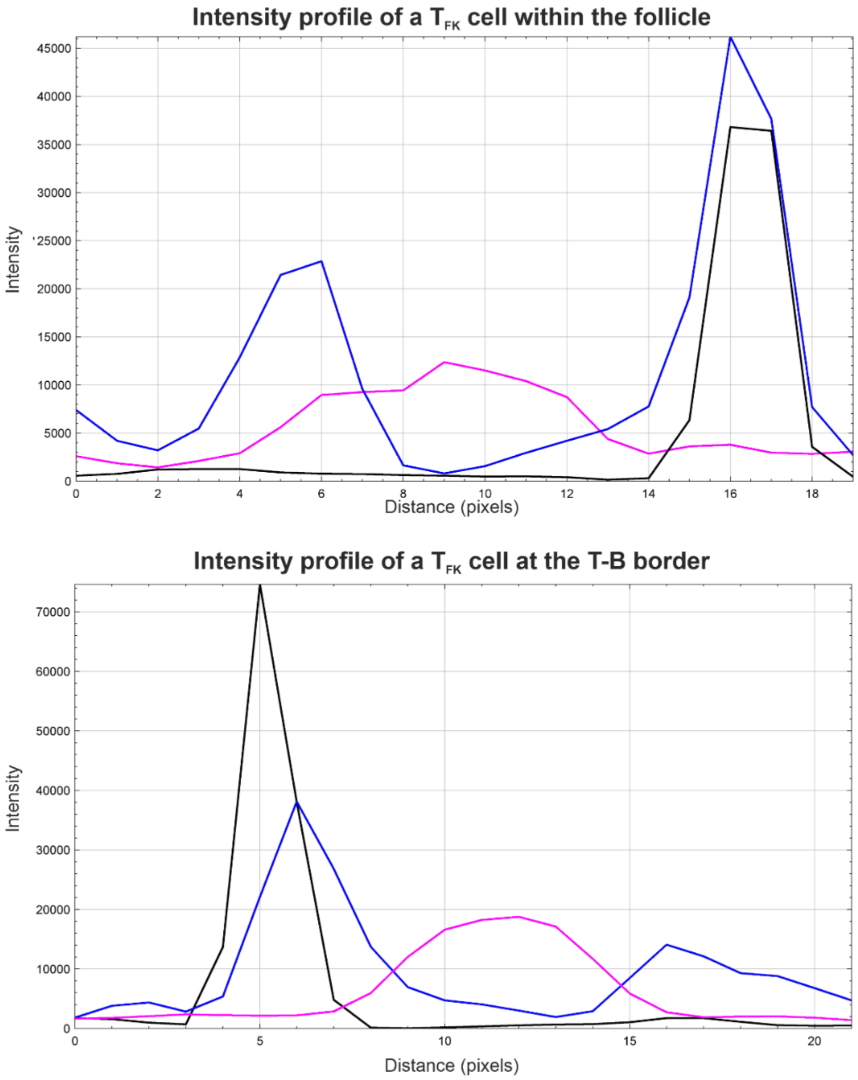

Supplementary Fig. 10: Representative intensity profiles of T_{FK} cells within a follicle compared to a T_{FK} cell located at the T-B border. Black: TIA-1, blue: CD4, magenta: BCL-6. In order to be confirmed as a T_{FK} cell, the intensity profile must overlap between CD4 and TIA-1 on the cell membrane while BCL-6 is only present in the nucleus. This means that the rise of intensity of BCL-6 is framed by two intensity peaks of CD4 while one or even both intensity peaks of CD4 coincide with an intensity peak of TIA-1.

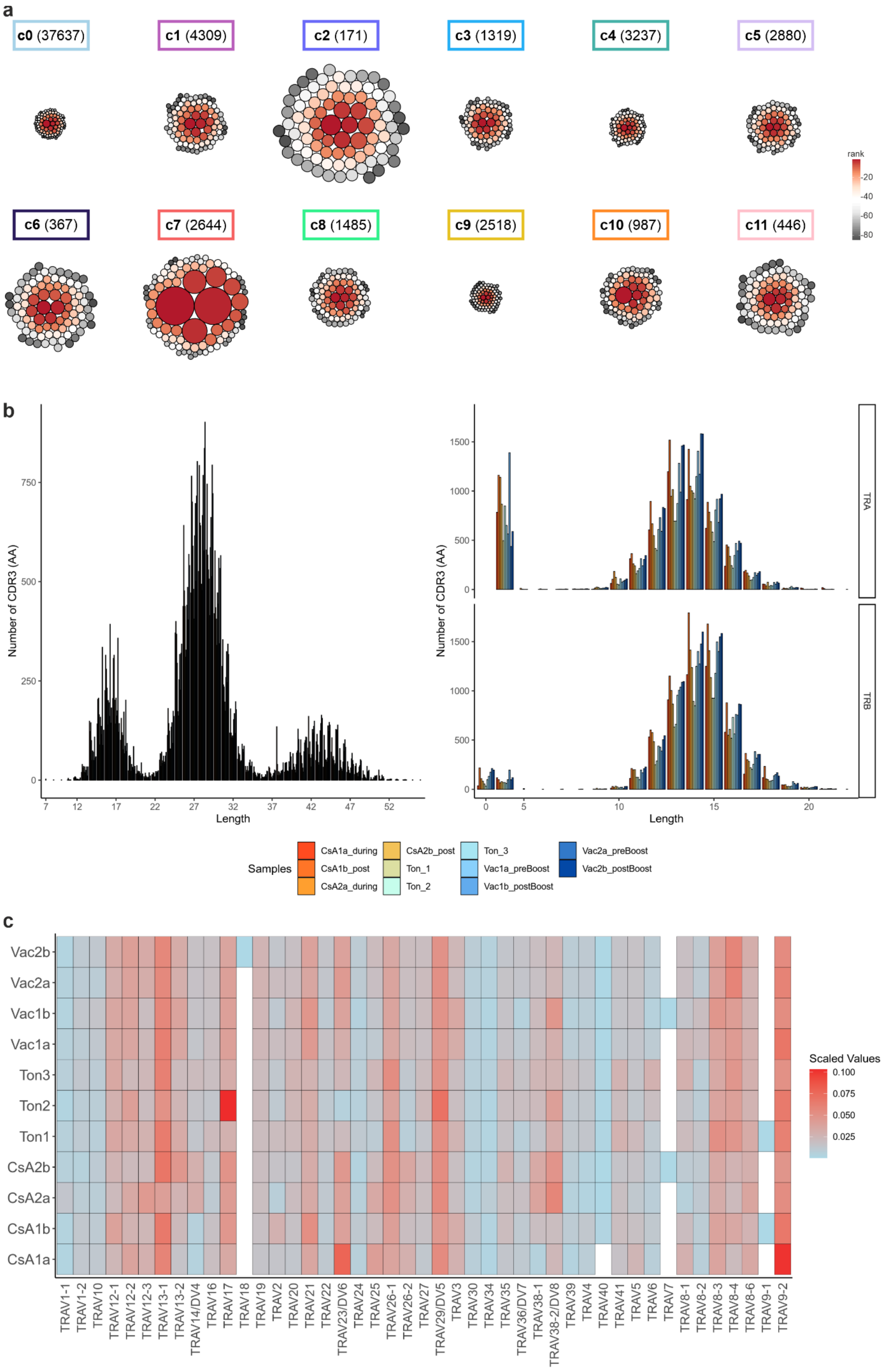
a
c0 (37637)
c1 (4309)
c2 (171)
c3 (1319)
c4 (3237)
c5 (2880)
c6 (367)
c7 (2644)
c8 (1485)
c9 (2518)
c10 (987)
c11 (446)
rank
20
40
60
80
b
Number of CDR3 (AA)
Length
TRA
TRB
Samples
CsA1a_during
CsA1b_post
CsA2a_during
CsA2b_post
Ton_1
Ton_2
Ton_3
Vac1a_preBoost
Vac1b_postBoost
Vac2a_preBoost
Vac2b_postBoost
c
Vac2b
Vac2a
Vac1b
Vac1a
Ton3
Ton2
Ton1
CsA2b
CsA2a
CsA1b
CsA1a
Scaled Values
0.100
0.075
0.050
0.025
TRAV1-1
TRAV1-2
TRAV10
TRAV12-1
TRAV12-2
TRAV12-3
TRAV13-1
TRAV13-2
TRAV14/DV4
TRAV16
TRAV17
TRAV18
TRAV19
TRAV2
TRAV20
TRAV21
TRAV22
TRAV23/DV6
TRAV24
TRAV25
TRAV26-1
TRAV26-2
TRAV27
TRAV29/DV5
TRAV3
TRAV30
TRAV34
TRAV35
TRAV36/DV7
TRAV38-1
TRAV38-2/DV8
TRAV39
TRAV4
TRAV40
TRAV41
TRAV5
TRAV6
TRAV7
TRAV8-1
TRAV8-2
TRAV8-3
TRAV8-4
TRAV8-6
TRAV9-1
TRAV9-2

Supplementary Fig. 11: C7 contains the largest T-cell clones. a Bubble plots visualize the top 85 clonotypes per cluster. The size of the circles is determined by the frequency of cells representing a clone of the cluster and relative to the cluster size, while the proportions of the clusters are consistent. The color gradient indicates the rank of a clone in terms of frequency per cluster (1-85). The box above each plot demonstrates the cluster represented next to the number of cells included. **b** Distribution of CDR3 lengths is illustrated in the bar plot (left), the length distribution of individual chains is shown separately in the right panel. **c** Comparison of TRAV gene usages in different samples illustrated in a heatmap.

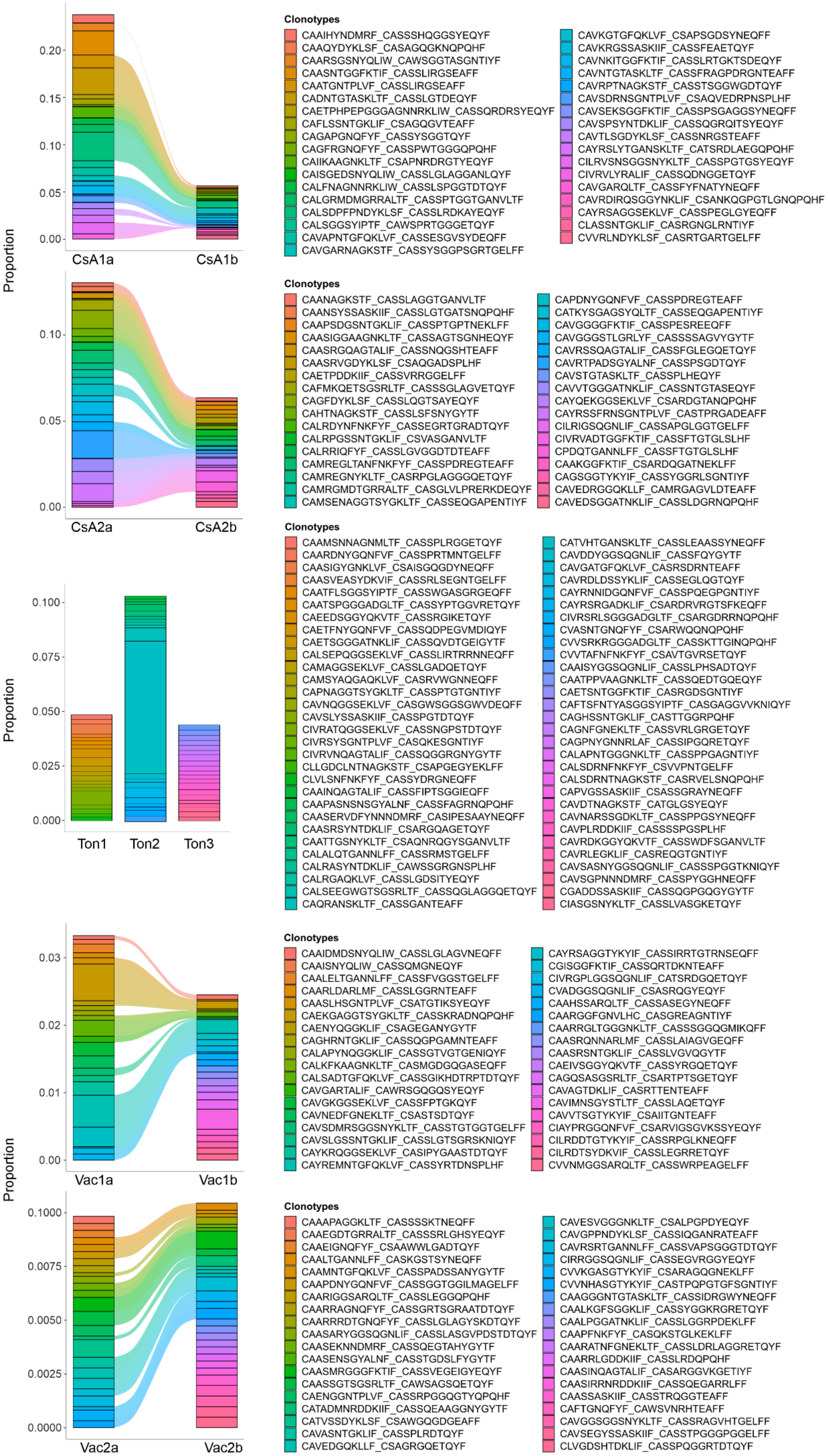
Proportion
0.20
0.15
0.10
0.05
0.00
CsA1a
CsA1b
Clonotypes
CAAIHYNDMRF_CASSSHQGGGSYEQYF
CAAQYDYKLSF_CASAGQGKNQPQHF
CAARSGSNYQLIW_CAWSGGTASGNTIYF
CAASNTGGFKTIF_CASSLIRGSEAFF
CAATGNTPLVF_CASSLIRGSEAFF
CADNTGTASKLTF_CASSLGTDEQYF
CAETPHPEPGGGAGNNRKLIW_CASSQRDRSYEQYF
CAFLSSNTGKLIF_CASAGQGVTEAFF
CAGAPGNQFYF_CASSYSGGTQYF
CAGFRGNQFYF_CASSPWTGGGQPQHF
CAIIKAAGNKLTF_CSAPNRDRGTYEQYF
CAISGEDSNYQLIW_CASSLGLAGGANLQYF
CALFNAGNNRKLIW_CASSLSPGGTDTQYF
CALGRMDMGRRALTF_CASSPTGGTGANVLTF
CALSDPFPNDYKLSF_CASSLRDKAYEQYF
CALSGGSYIPTF_CAWSPRTGGGETQYF
CAVAPNTGFQKLVF_CASESGVSYDEQFF
CAVGARNAGKSTF_CASSYSGGPSGRTGELFF
CAVKGTGFQKLVF_CSAPSGDSYNEQFF
CAVKRGSSASKIIF_CASSFEAETQYF
CAVNKITGGFKTIF_CASSLRTGKTSDEQYF
CAVNTGTASKLTF_CASSFRAGPDRGNTEAFF
CAVRPTNAGKSTF_CASSTSGGWGDTQYF
CAVSDRNSGNTPLVF_CSAQVEDRPNSPLHF
CAVSEKSGGFKTIF_CASSPSGAGGSYNEQFF
CAVSPSYNTDKLIF_CASSQGRQITSYEQYF
CAVTLSGDYKLSF_CASSNRGSTEAFF
CAYRSLYTGANSKLTF_CATSRDLAEGQPQHF
CILRVSNSGGSNYKLTF_CASSPGTGSYEQYF
CIVRVLYRALIF_CASSQDNGGETQYF
CAVGARQLTF_CASSFYFNATYNEQFF
CAVRDIRQSGGYNKLIF_CSANKQGPGTLGNQPQHF
CAYRSAGGSEKLVF_CASSPEGLGYEQFF
CLASSNTGKLIF_CASRGNGLRNTIYF
CVVRLNDYKLSF_CASRTGARTGELFF
Proportion
0.10
0.05
0.00
CsA2a
CsA2b
Clonotypes
CAANAGKSTF_CASSLAGGTGANVLTF
CAANSYSSASKIIF_CASSLGTGATSNQPQHF
CAAPSDGSNTGKLIF_CASSPTGPTNEKLFF
CAASIGGAAGNKLTF_CASSAGTSGNHEQYF
CAASRGQAGTALIF_CASSNQGSHTEAFF
CAASRVGDYKLSF_CSAQGADSPLHF
CAETPDDKIIF_CASSVRRGGELFF
CAFMKQETSGSRLTF_CASSSGLAGVETQYF
CAGFDYKLSF_CASSLQGTSAYEQYF
CAHTNAGKSTF_CASSLSFSNYGYTF
CALRDYNFNKFYF_CASSEGRTGRADTQYF
CALRPGSSNTGKLIF_CSVASGANVLTF
CALRRIQFYF_CASSLGVGGDTDTEAFF
CAMREGLTANFNKFYF_CASSPDREGTEAFF
CAMREGNYKLTF_CASRPGLAGGGQETQYF
CAMRGMDTGRRALTF_CASGLVLPRERKDEQYF
CAMSENAGGTSYGKLTF_CASSEQGAPENTIYF
CAPDNYGQNFVF_CASSPDREGTEAFF
CATKYSGAGSYQLTF_CASSEQGAPENTIYF
CAVGGGGFKTIF_CASSPESREEQFF
CAVGGGSTLGRLYF_CASSSSAGVYGYTF
CAVRSSQAGTALIF_CASSFGLEGQETQYF
CAVRTPADSGYALNF_CASSPSGDTQYF
CAVSTGTASKLTF_CASSPLHEQYF
CAVVTGGGATNKLIF_CASSNTGTASEQYF
CAYQEKGGSEKLVF_CSARDGTANQPQHF
CAYRSSFRNSGNTPLVF_CASTPRGADEAFF
CILRIGSQGNLIF_CASSAPGLGGTGELFF
CIVRVADTGGFKTIF_CASSFTGTGLSLHF
CPDQTGANNLFF_CASSFTGTGLSLHF
CAAKGGFKTIF_CSARDQGATNEKLFF
CAGSGGTYKYIF_CASSYGGRLSGNTIYF
CAVEDRGGQKLLF_CAMRGAGVLDTEAFF
CAVEDSGGATNKLIF_CASSLDGRNQPQHF
Clonotypes
Proportion
0.100
0.075
0.050
0.025
0.000
Ton1
Ton2
Ton3
CAAMSNNAGNMLTF_CASSPLRGGETQYF
CAARDNYGQNFVF_CASSPRTMNTGELFF
CAASIGYGNKLVF_CSAISGQGDYNEQFF
CAASVEASYDKVIF_CASSRLSEGNTGELFF
CAATFLSGGSYIPTF_CASSWGASGRGEQFF
CAATSPGGGADGLTF_CASSYPTGGVRETQYF
CAEEDSGGYQKVTF_CASSRGIKETQYF
CAETFNYGQNFVF_CASSQDPEGVMDIQYF
CAETSGGGATNKLTF_CASSQVDTGEIGYTF
CALSEPQGGSEKLVF_CASSLIRTRRNNEQFF
CAMAGGSEKLVF_CASSLGADQETQYF
CAMSYAQGAQKLVF_CASRVWGNNEQFF
CAPNAGGTSYGKLTF_CASSPTGTGNTIYF
CAVNQGGSEKLVF_CASGWSGGSGWVDEQFF
CAVSLYSSASKIIF_CASSPGTDTQYF
CIVRATQGGSEKLVF_CASSNGPSTDTQYF
CIVRSYSGNTPLVF_CASQKESGNTIYF
CIVRVNQAGTALIF_CASSQGGRGNYGYTF
CLLGDCLNTNAGKSTF_CSAPGEGYEKLFF
CLVLSNFNKFYF_CASSYDRGNEQFF
CAAINQAGTALIF_CASSFIPTSGGIEQFF
CAAPASNSNSGYALNF_CASSFAGRNQPQHF
CAASERVDFYNNNDMRF_CASIPESAAYNEQFF
CAASRSYNTDKLIF_CASRGQAGETQYF
CAATTGSNYKLTF_CSAQNRQGYSGANVLTF
CALALQTGANNLFF_CASSRMSTGELFF
CALRASYNTDKLIF_CAWSSGRGNSPLHF
CALRGAQKLVF_CASSLGDSITYEQYF
CALSEEGWGTSGSRLTF_CASSQGLAGGQETQYF
CAQRANSKLTF_CASSGANTEAFF
CATVHTGANSKLTF_CASSLEAASSYNEQFF
CAVDDYGGSQGNLIF_CASSFQYGYTF
CAVGATGFQKLVF_CASRSDRNTEAFF
CAVRDLDSSYKLIF_CASSEGLQGTQYF
CAYRNNIDGQNFVF_CASSPQEGPGNTIYF
CAYRSRGADKLIF_CSARDRVRGTSFKEQFF
CIVRSRLSGGGADGLTF_CSARGDRRNQPQHF
CVASNTGNQFYF_CSARWQQNQPQHF
CVVSRKRGGGADGLTF_CASSKTTGINQPQHF
CVVTAFNFNKFYF_CSAVTGVRSETQYF
CAAISYGGSQGNLIF_CASSLPHSADTQYF
CAATPPVAAGNKLTF_CASSQEDTGQEQYF
CAETSNTGGFKTIF_CASRGDSGNTIYF
CAFTSFNTYASGGSYIPTF_CASGAGGVVKNIQYF
CAGHSSNTGKLIF_CASTTGGRPQHF
CAGNFGNEKLTF_CASSVRLGRGETQYF
CAGPNYGNNRLAF_CASSIPGQRETQYF
CALAPNTGGGNKLTF_CASSPPGAGNTIYF
CALSDRNFNKFYF_CSVVPNTGELFF
CALSDRNTNAGKSTF_CASRVELSNQPQHF
CAPVGSSASKIIF_CSASSGRAYNEQFF
CAVDTNAGKSTF_CATGLGSYEQYF
CAVNARSSGDKLTF_CASSPPGSYNEQFF
CAVPLRDDKIIF_CASSSSPGSPLHF
CAVRDKGGYQKVTF_CASSWDFSGANVLTF
CAVRLEGKLIF_CASREQGTGNTIYF
CAVSASNYGGSQGNLIF_CASSSPGGTKNIQYF
CAVSGPNNNDMRF_CASSPYGGHNEQFF
CGADDSSASKIIF_CASSQGPGQGYGYTF
CIASGSNYKLTF_CASSLVASGKETQYF
Proportion
0.03
0.02
0.01
0.00
Vac1a
Vac1b
Clonotypes
CAAIDMDSNYQLIW_CASSLGLAGVNEQFF
CAAISNYQLIW_CASSQMGNEQYF
CAALELTGANNLFF_CASSFVGGSTGELFF
CAARLDARLMF_CASSLGGRNTEAFF
CAASLHSGNTPLVF_CSATGTIKSYEQYF
CAEKGAGGTSYGKLTF_CASSKRADNQPQHF
CAENYQGGKLIF_CSAGEGANYGYTF
CAGHRNTGKLIF_CASSQGPGAMNTEAFF
CALAPYNQGGKLIF_CASSGTVGTGENIQYF
CALKFKAAGNKLTF_CASMGDGQGASEQFF
CALSADTGFQKLVF_CASSGIKHDTRPTDTQYF
CAVGARTALIF_CAWRSGQGQSYEQYF
CAVGKGGSEKLVF_CASSFPTGKQYF
CAVNEDFGNEKLTF_CSATSTSDTQYF
CAVSDMRSGGSNYKLTF_CASSTGTGGTGELFF
CAVSLGSSNTGKLIF_CASSLGTSGRSKNIQYF
CAYKRQGGSEKLVF_CASIPYGAASTDTQYF
CAYREMNTGFQKLVF_CASSYRTDNSPLHF
CAYRSAGGTYKYIF_CASSIRRTGTRNSEQFF
CGISGGFKTIF_CASSQRTDKNTEAFF
CIVRGPLGGSQGNLIF_CATSRDGQETQYF
CVADGGSQGNLIF_CSASRQGYEQYF
CAAHSSARQLTF_CASSASEGYNEQFF
CAARGGFGNVLHC_CASGREAGNTIYF
CAARRGLTGGGNKLTF_CASSSGGQGMIKQFF
CAASRQNNARLMF_CASSLAIAGVGEQFF
CAASRSNTGKLIF_CASSLVGVQGYTF
CAEIVSGGYQKVTF_CASSYRGQETQYF
CAGQSASGSRLTF_CSARTPTSGETQYF
CAVAGTDKLIF_CASRTTENTEAFF
CAVIMNSGYSTLTF_CASSLAQETQYF
CAVVTSGTYKYIF_CSAIITGNTEAFF
CIAYPRGGQNFVF_CSARVIGSGVKSSYEQYF
CILRDDTGTYKYIF_CASSRPGLKNEQFF
CILRDTSYDKVIF_CASSLEGRRETQYF
CVVNMGGSARQLTF_CASSWRPEAGELFF
0.1000
0.0075
0.0050
0.0025
0.0000
Vac2a
Vac2b
Clonotypes
CAAAPAGGKLTF_CASSSSKTNEQFF
CAAEGDTGRRALTF_CASSSRLGHSYEQYF
CAAEIGNQFYF_CSAAWWLGADTQYF
CAALTGANNLFF_CASKGSTSYNEQFF
CAAMNTGFQKLVF_CASSPADSSANYGYTF
CAAPDNYGQNFVF_CASSGGTGGILMAGELFF
CAARIGGSARQLTF_CASSLEGGQPQHF
CAARRAGNQFYF_CASSGRTSGRAATDTQYF
CAARRRDTGNQFYF_CASSLGLAGYSKDTQYF
CAASARYGGSQGNLIF_CASSLASGVPDSTDTQYF
CAASEKNNDMRF_CASSQEGTAHYGYTF
CAASENSGYALNF_CASSTGDSLFYGYTF
CAASMRGGGFKTIF_CASSVEGEIGYEQYF
CAASSGTSGSRLTF_CAWSAGSQETQYF
CAENGGNTPLVF_CASSRPGGQGTYQPQHF
CATADMNRDDKIIF_CASSQEAAGGNYGYTF
CATVSSDYKLSF_CSAWGQGDGEAFF
CAVASNTGKLIF_CASSPLRDTQYF
CAVEDGQKLLF_CSAGRGQETQYF
CAVESVGGGNKLTF_CSALPGPDYEQYF
CAVGPPNDYKLSF_CASSIQGANRATEAFF
CAVRSRTGANNLFF_CASSVAPSGGGTDTQYF
CIRRGGSQGNLIF_CASSEGVRGGYEQYF
CVVKGASGTYKYIF_CSARAGQGNEKLFF
CVVNHASGTYKYIF_CASTPQPGTGFSGNTIYF
CAAGGGNTGTASKLTF_CASSIDRGWYNEQFF
CAALKGFSGGKLIF_CASSYGGKRGRETQYF
CAALPGGATNKLIF_CASSLGGRPDEKLFF
CAAPFNKFYF_CASQKSTGLKEKLFF
CAARATNFGNEKLTF_CASSLDRLAGGRETQYF
CAARRLGDDKIIF_CASSLRDQPQHF
CAASINQAGTALIF_CASARGGVKGETIYF
CAASIRRNRDDKIIF_CASSQEGARRLFF
CAASSASKIIF_CASSTRQGGTEAFF
CAFTGNQFYF_CAWSVNRHTEAFF
CAVGGSGGSNYKLTF_CASSRAGVHTGELFF
CAVSEGYSSASKIIF_CASSTPGGGPGGELFF
CLVGDSHTDKLIF_CASSPQGGRTDTQYF

Supplementary Fig. 12: Proportion of TCR clones. Their persistence upon treatment and the sequences of the top clonotypes per cell origin. The top 30 clonotypes of all PB donor groups or the top 60 of the tonsils are indicated in bars, the size represents the proportion in the total types.
