# Supplementary material for: Oligoclonal CD4+CXCR5+ T cells with a cytotoxic phenotype appear in tonsils and blood
Source: Commun Biol. 2024 Jul 18;7:879. doi: 10.1038/s42003-024-06563-1 (PMC11258247; doi:10.1038/s42003-024-06563-1)
Supplement: Supplementary file 3 — Description of Additional Supplementary Files [file 42003_2024_6563_MOESM3_ESM.pdf]

Description of Additional Supplementary Files

File name: Supplementary Data 1
Description: 50 differentially expressed genes of each cluster.

File name: Supplementary Data 2
Description: The source data behind the graphs in the paper.
