# Supplementary material for: Oligoclonal CD4+CXCR5+ T cells with a cytotoxic phenotype appear in tonsils and blood
Source: Commun Biol. 2024 Jul 18;7:879. doi: 10.1038/s42003-024-06563-1 (PMC11258247; doi:10.1038/s42003-024-06563-1)
Supplement: Supplementary file 6 — Reporting summary [file 42003_2024_6563_MOESM6_ESM.pdf]

nature portfolio

Corresponding author(s): Friederike Berberich-Siebelt

Last updated by author(s): Jun 24, 2024

Reporting Summary

Nature Portfolio wishes to improve the reproducibility of the work that we publish. This form provides structure for consistency and transparency in reporting. For further information on Nature Portfolio policies, see our Editorial Policies and the Editorial Policy Checklist.

Statistics

For all statistical analyses, confirm that the following items are present in the figure legend, table legend, main text, or Methods section.

n/a	Confirmed	
☐	☒	The exact sample size (*n*) for each experimental group/condition, given as a discrete number and unit of measurement
☐	☒	A statement on whether measurements were taken from distinct samples or whether the same sample was measured repeatedly
☐	☒	The statistical test(s) used AND whether they are one- or two-sided<br>*Only common tests should be described solely by name; describe more complex techniques in the Methods section.*
☐	☒	A description of all covariates tested
☐	☒	A description of any assumptions or corrections, such as tests of normality and adjustment for multiple comparisons
☐	☒	A full description of the statistical parameters including central tendency (e.g. means) or other basic estimates (e.g. regression coefficient) AND variation (e.g. standard deviation) or associated estimates of uncertainty (e.g. confidence intervals)
☒	☐	For null hypothesis testing, the test statistic (e.g. *F*, *t*, *r*) with confidence intervals, effect sizes, degrees of freedom and *P* value noted<br>*Give P values as exact values whenever suitable.*
☒	☐	For Bayesian analysis, information on the choice of priors and Markov chain Monte Carlo settings
☒	☐	For hierarchical and complex designs, identification of the appropriate level for tests and full reporting of outcomes
☒	☐	Estimates of effect sizes (e.g. Cohen's *d*, Pearson's *r*), indicating how they were calculated

Our web collection on statistics for biologists contains articles on many of the points above.

Software and code

Policy information about availability of computer code

Data collection	Freshly sorted human TFH cells were loaded on the Chromium Single Cell Controller (10x Genomics) using the Single Cell 5′ Library & Gel Bead Kit v1.1 (10x Genomics). Single Cell V(D)J Enrichment Kit, Human T Cell (10x Genomics) (10 cycles of PCR). Raw data were processed using Cellranger to complete the data collection.
Data analysis	Seurat 4, scRepertoire, ggplot2, velocyto, scVelo, packcircles, R

For manuscripts utilizing custom algorithms or software that are central to the research but not yet described in published literature, software must be made available to editors and reviewers. We strongly encourage code deposition in a community repository (e.g. GitHub). See the Nature Portfolio guidelines for submitting code & software for further information.

Data

Policy information about availability of data

All manuscripts must include a data availability statement. This statement should provide the following information, where applicable:

- Accession codes, unique identifiers, or web links for publicly available datasets
- A description of any restrictions on data availability
- For clinical datasets or third party data, please ensure that the statement adheres to our policy

Sequencing read counts after mapping and filtering are deposited with (GEO accession number GSE218131 for scRNAseq, CITEseq and TCRseq). Due to privacy and ethical concerns, the raw sequencing data cannot be made available.

Research involving human participants, their data, or biological material

Policy information about studies with human participants or human data. See also policy information about sex, gender (identity/presentation), and sexual orientation and race, ethnicity and racism.

Reporting on sex and gender	Based on self-reports, an equal number (one male, one female CsA-treated allo-HSCT patients, one male, one female vaccinee, one male, two female tonsils, so far all for sequencing, further four male and four female tonsils for flow cytometry) of male and female donors were included. Informed consent was given in general, not in particular about sharing sexes. No informatin was available about blood samples from healthy donors for flow cytometry.
Reporting on race, ethnicity, or other socially relevant groupings	No big cohorts comparing race, ethniciy or other socially relevant groupings could be included in this study. All (or most; no information about blood donors) participants were Caucasian.
Population characteristics	Please see details about healthy and patient donors in Supplementary Table 1.
Recruitment	Subjects were recruited by investigators certified to conduct clinical research at the University Hospital of Erlangen.
Ethics oversight	Ethical approval was given by the ethical board of the University of Erlangen (proposal numbers 3762, 444_19 B, 173_17 B.

Note that full information on the approval of the study protocol must also be provided in the manuscript.

Field-specific reporting

Please select the one below that is the best fit for your research. If you are not sure, read the appropriate sections before making your selection.

☒ Life sciences ☐ Behavioural & social sciences ☐ Ecological, evolutionary & environmental sciences

For a reference copy of the document with all sections, see nature.com/documents/nr-reporting-summary-flat.pdf

Life sciences study design

All studies must disclose on these points even when the disclosure is negative.

Sample size	Sample sizes were maximized based on availability of clinical samples. and possiblity to process them. No prospective sample size calculations were performed.
Data exclusions	No data very excluded, except cells lacking the combined information of TCRα and TCRβ chain, those CDR3 nucleotide sequence were excluded from analysis.
Replication	For single cell sequencing, all probes were run in parallel. However, each condition was represented twice or trice. Data were verified with other methods until reaching statistically significant values.
Randomization	not relevant
Blinding	Blinding was not possible during analysis since comparisons were based on type of sample like blood versus tonsil. However, within one group, for example healthy volunteers or tonsil donors, the analysist was not aware of medical differences.

Reporting for specific materials, systems and methods

We require information from authors about some types of materials, experimental systems and methods used in many studies. Here, indicate whether each material, system or method listed is relevant to your study. If you are not sure if a list item applies to your research, read the appropriate section before selecting a response.

Materials & experimental systems

n/a	Involved in the study	
☐	☒	Antibodies
☒	☐	Eukaryotic cell lines
☒	☐	Palaeontology and archaeology
☒	☐	Animals and other organisms
☐	☒	Clinical data
☒	☐	Dual use research of concern
☒	☐	Plants

Methods

n/a	Involved in the study	
☒	☐	ChIP-seq
☐	☒	Flow cytometry
☒	☐	MRI-based neuroimaging

Antibodies

Antibodies used

flow cytometric sorting:
CD3 SK7 BV510 BD Biosciences
CD4 SK3 PE BD Biosciences
CD45RA L48 FITC BD Biosciences
CXCR5 RF8B2 AF647 BD Biosciences
CD25 BC96 TotalSeq™-C0085 BioLegend
CD279 EH122H7 TotalSeq™- C0088 BioLegend
CD183 G025H7 TotalSeq™-C00140 BioLegend
CD196 G034E3 TotalSeq™-C00143 BioLegend

flow cytometric analyses:
CD3 SK7 BUV395 BD Biosciences
CD3 OKT3 AF700 BioLegend
CD4 SK3 FITC BioLegend
CD4 SK3 Spark NIR 685 BioLegend
CD8a SK1 BUV805 BD Biosciences
CD8b REA715 VioGreen Miltenyi Biotec
CD19 SJ25C1 BUV737 BD Biosciences
CD45RA HI100 BV711 BD Biosciences
CD45RA HI100 PerCP BioLegend
CD56 NCAM16.2 BUV615 BD Biosciences
CD107a H4A3 BV786 BD Biosciences
CXCR5 RF8B2 AF647 BD Biosciences
CXCR5 J252D4 APC BioLegend
EOMES WD1928 PE-Cy7 Invitrogen, Thermo Fisher
Granzyme B GB11 BV421 BD Biosciences
TIA-1 2G9 PE Beckman Coulter
PD-1 EH12.2H7 Dazzle 594 BioLegend
CXCR3 G025H7 BV510 BioLegend
CCR6 G034E3 BV650 BioLegend
CD57 QA17A04 BV711 BioLegend

IF histology staining:
CD4 AF-379-NA 1:150 R&D
TIA-1 IM2550 1:200 Beckman Coulter
BCL-6 14859 1:50 Cell signaling
CD19 14-0194-82 1:500 Invitrogen
IgD 2032-01 1:100 Southern Biotech
CD23 NCL-L-CD23-1B12 1:250 Leica
Ki67 14-5698-82 1:200 Invitrogen
CXCR5 EPR23463-30 1:300 Abcam
Hoechst - 1:50000 Sigma (#B2261)
donkey anti-goat ab150132 Alexa Fluor 594 Abcam
donkey anti-rabbit A31572 Alexa Fluor 555 ThermoFisher
donkey anti-rat A21208 Alexa Fluor 488 ThermoFisher
donkey anti-mouse A31573 Alexa Fluor 647 ThermoFisher
donkey anti-goat A11056 Alexa Fluor 546 ThermoFisher

Validation

All antibodies were used according to the manufacturer's instructions and validated using healthy donor human PBMCs.

Clinical data

Policy information about clinical studies
All manuscripts should comply with the ICMJE guidelines for publication of clinical research and a completed CONSORT checklist must be included with all submissions.

Clinical trial registration

Provide the trial registration number from ClinicalTrials.gov or an equivalent agency.

Study protocol

Note where the full trial protocol can be accessed OR if not available, explain why.

Data collection

Describe the settings and locales of data collection, noting the time periods of recruitment and data collection.

Outcomes

Describe how you pre-defined primary and secondary outcome measures and how you assessed these measures.

Plants

Seed stocks

Report on the source of all seed stocks or other plant material used. If applicable, state the seed stock centre and catalogue number. If plant specimens were collected from the field, describe the collection location, date and sampling procedures.

Novel plant genotypes

Describe the methods by which all novel plant genotypes were produced. This includes those generated by transgenic approaches, gene editing, chemical/radiation-based mutagenesis and hybridization. For transgenic lines, describe the transformation method, the number of independent lines analyzed and the generation upon which experiments were performed. For gene-edited lines, describe the editor used, the endogenous sequence targeted for editing, the targeting guide RNA sequence (if applicable) and how the editor was applied.

Authentication

Describe any authentication procedures for each seed stock used or novel genotype generated. Describe any experiments used to assess the effect of a mutation and, where applicable, how potential secondary effects (e.g. second site T-DNA insertions, mosiacism, off-target gene editing) were examined.

Flow Cytometry

Plots

Confirm that:

☒ The axis labels state the marker and fluorochrome used (e.g. CD4-FITC).

☒ The axis scales are clearly visible. Include numbers along axes only for bottom left plot of group (a 'group' is an analysis of identical markers).

☒ All plots are contour plots with outliers or pseudocolor plots.

☒ A numerical value for number of cells or percentage (with statistics) is provided.

Methodology

Sample preparation

Patient and healthy donor samples. Tonsils were taken from donors with no, mild, and recurrent / moderate tonsillitis (Supplementary Table 1). cTFH were isolated from PBMCs of healthy volunteers before and after medically indicated routine booster vaccination (tetanus toxoid) in healthy individuals and PBMCs from patients before and after tapering of cyclosporin A (CsA) following allogeneic hematopoietic stem cell transplantation (allo-HSCT). For the Study Design, see Supplementary Fig. 1a. Further PB was donated by healthy volunteers.

Flow cytometric cell sorting for transcriptome and TCR repertoire analysis. Cryopreserved samples were rapidly thawed in a water bath at 37°C, diluted with warm RPMI 1640 (BioWhittaker, Lonza, Walkersville, MD, USA)/10% FCS/10mM HEPES to 10 ml, centrifuged (300 x g, 10 min) and either counted (tonsils) or directly resuspended in 100 µl FACS buffer (PBS/2% FCS). Cells (approx. 5x106) were stained with fluorochrome- and oligo-coupled antibodies (Supplementary Table 3) for 20 min at 4° C, washed once and resuspended in 900 µl FACS buffer. Immediately prior to sorting, DAPI (Sigma-Aldrich, Merck, Darmstadt, Germany) was added for dead cell exclusion. Cells were sorted into 1.5 ml DNA LoBind tubes (Eppendorf, Hamburg, Germany) pre-filled with 0.5 ml PBS/10% FCS and directly processed for molecular analysis.

scRNAseq, scTCRseq and scCITEseq library preparation and sequencing. Freshly sorted human TFH cells were loaded on the Chromium Single Cell Controller (10x Genomics) using the Single Cell 5′ Library & Gel Bead Kit v1.1 (10x Genomics #PN-1000165). cDNAs were amplified using 13-14 cycles of PCR. ScRNAseq libraries were constructed using 14-15 cycles of PCR and scCITEseq libraries were constructed using 9 cycles of PCR. ScTCRseq libraries were generated from the same cDNAs using the Single Cell V(D)J Enrichment Kit, Human T Cell (10x Genomics) (10 cycles of PCR). Products were purified using Ampure XP beads and quality was controlled using Agilent Tapestation.

Flow cytometric analysis of human MNC from peripheral blood and tonsils. PBMCs were isolated from freshly drawn blood samples by density gradient centrifugation over Ficoll (Pancoll human, PAN BIOTECH, Aidenbach, Germany). Frozen tonsil MNC were rapidly thawed (see above). Staining was performed in PBS/2% FCS (1x106 cells/100 µl) and brilliant stain buffer (BD Biosciences, Heidelberg, Germany). For dead cell exclusion, fixable viability dye (Zombie UVTM, BioLegend, San Diego, CA, USA) was added prior to staining. For staining of intracellular markers, the eBioscience Foxp3/Transcription Factor Staining Buffer Set (Invitrogen by Thermo Fisher Scientific, Carlsbad, CA, USA) was used.

Immunofluorescence (IF) histology staining. Consecutive formalin-fixed paraffin embedded (FFPE) sections were used for localization of TFK cells. Deparaffinized FFPE sections, 3-4 µm from each embedded block, underwent heat-induced antigen retrieval (20 mM citric acid buffer, pH 6.0). Sections were blocked with Antibody Diluent (Dako, #S3022) for 1 h at RT before incubation with primary antibodies (Supplementary Table 5) in Antibody Diluent for 1 h. For thoroughly washed sections (3x with TBST) secondary antibodies (1:1000) were prepared in PBS containing 0.05% Tween 20 (Sigma, P9416-50ML) and Hoechst for 1 h at RT. After washing with TBST, sections were embedded in Fluoromount-G Mounting medium (ThermoFisher, #00-4958-02).

Instrument

Sorting was performed on a FACSAriaTM Fusion highspeed cell sorter (4-way purity; 85 µm nozzle; BD Biosciences); gating strategy in Supplementary Fig. 1b, c.

ScRNA- and scCITEseq libraries were paired-end sequenced (S1 flow cell, 100 bp) on the Illumina NovaSeq 6000™. ScTCRseq libraries were single end sequenced (High Output flow cell, 150 cycles) on the Illumina NextSeq 550.

Flow cytometry data were acquired on a FACSymphonyTM A5 (BD Biosciences).

Representative IF images were acquired at confocal laser-scanning microscope Zeiss LSM780. Plan-Apochromat 20 × 0,5 and C-Apochromat 40x 1.2 W objectives were used for detection in four simultaneous channels.

Software

Single-cell transcriptome profiling. Cellranger 6.1 was used to align the sequencing reads to a reference human genome to generate the counting matrix. Data analysis was performed using Seurat 4 77. Cells of low quality defined by high level of mitochondrial gene expression (> 20%) were filtered out, in addition, only cells of gene feature number ranging between 300 and 3500 were kept for the following analysis. Here we excluded one gene feature, XIST to avoid the bias introduced by the donor gender. Batch effects were initially removed with the Seurat integrated canonical correlation analysis (CCA). Anchor genes were computed, which enabled precise data integration and cell clustering. Afterwards cell clusters were identified by a Seurat function "FindCluster", where a resolution parameter 0.5 was specified and the Louvain algorithm was applied. The cluster annotation was carried out with SingleR (version: 1.8) package 78 and PanglaoDB 79. ADT de-multiplexing and analysis were performed in Seurat, normalization method CLR. A non-linear dimension reduction for visualization was achieved by UMAP.

Single-cell TCR repertoire profiling. The cellranger vdj pipeline (version 6.1) was applied to count T-cell receptor sequencing data produced by VDJ libraries to get the profile, followed by a data analysis with R scRepertoire package 80. For the generation of bubble plots, TCR clonotypes were defined as cells expressing the same CDR3 nucleotide sequence in their TCRα and TCRβ chain, respectively. Cells lacking the combined information, CDR3 nucleotide sequence were excluded from analysis. For direct comparisons of clonality between groups, the number of considered cells per group was randomly reduced to the cell number of the smallest group. TCR clonality analysis was performed in R and visualized by using the packages packcircles and ggplot2.

RNAvelocity analysis. Splicing information was collected with velocyto, examining all the bam files generated by cellranger. Results were imported into scVelo for downstream RNAvelocity analysis 81. The figure was plotted in a grid and stream style implemented in the scVelo.

Flow cytometry data were analyzed with FlowJo® v10.8.1 (Treestar Inc., Ashland, OR, USA).

IF pictures were evaluated using the software Fiji (ImageJ) 82.

Cell population abundance

Sorted CD4+CD45RA-CXCR5+ comprised approximately 2 % of input cells.

Gating strategy

For Flow Cytometry analysis: SSC-A/FSC-A were used for lymphocytes gating; FSC-H/FSC-A were used for single cells discrimination. CD3+ T cells were gated from live Zombie negative cells. CD4+ and CD8+ T cells were then gated and next CD16+ CCR6+ T cells. CD4+ FOXP3+ cells were then gated for identification of described CD4+ FOXP3+ CD56+ T cells and CD4 + FOXP3+ TIGIT- HLA-DR+ T cells.

☒ Tick this box to confirm that a figure exemplifying the gating strategy is provided in the Supplementary Information.
